# An explorative study of psychological and social factors impacting littering behavior in Vietnam

**DOI:** 10.3389/fpsyg.2022.1025062

**Published:** 2022-12-09

**Authors:** Anran Zhang, Bo Pang, Jeawon Kim, Tuyet-Mai Nguyen, Phong Tuan Nham

**Affiliations:** ^1^School of Economics and Management, Xidian University, Xi’an, China; ^2^Social Marketing @ Griffith, Griffith University, Brisbane, QLD, Australia; ^3^Department of Information and E-Commerce, Thuongmai University, Hanoi, Vietnam; ^4^School of Business Administration, VNU University of Economics and Business, Hanoi, Vietnam

**Keywords:** littering, connectedness to nature, social norms, environmental consciousness, intention to stop littering

## Abstract

Littering is a worldwide problem and Vietnam is one of the most affected countries. To enact change, not only individual cognitive determinants but also social and natural-, or environment-related variables should be taken into consideration. Although there is a large body of literature researching littering, most researchers do not distinguish the level of these factors. Thus, this research aims to investigate the interactive mechanism of these different level factors influencing the intention of the Vietnamese to stop littering, with the multi-level social-ecological model used to guide model building. The data were collected through a self-reported online questionnaire and the Partial Least Squares Structural Equation Modelling (PLS-SEM) method was employed to examine the proposed conceptual framework. The results indicated that perceived behavioral control and connectedness to nature are the two main factors influencing the Vietnamese people’s intention to stop littering. Multi-group analysis results suggested the moderating effects of injunctive and descriptive norms. This research proposed a new conceptual framework and achieved unique insights into littering behavior in Vietnam, which could benefit and guide behavioral change experts, academics, and practitioners to design appropriate marketing strategies/campaigns to reduce littering.

## Introduction

The world in the past few decades has been undergoing climate changes and other serious environmental alterations, which have threatened the Earth’s life-support system and human lives (Sachs, 2012). At the micro-level, littering is one of the problems hindering the sustainable development of the entire world because of its negative impacts on the environment, health, biodiversity, and even the economy (Chaudhary et al., 2021). The generation of sulfur oxides, nitrogen oxides, and carbon monoxide pollutes the air, which would further causes climate change (Thanh and Matsui, 2013). Besides, the spread of odors, flies, mosquitoes, rodents, and dust has detrimentally affected surrounding communities (World Bank Group, 2017). Cleaning up litter costs a significant amount of a city’s and/or a country’s budget, thus, preventing or reducing littering behavior or behavioral intention is important to solve this problem (Almosa et al., 2017a).

The social marketing approach—understanding behavior and designing interventions to promote voluntary change, was proven to be effective in influencing individual behavior and bringing about environmental, organizational, and even systemic change (Stead et al., 2007). As for littering, although there is a large body of literature researching littering [e.g., Cialdini et al., 1990; Schultz et al., 2013; Bateson et al., 2015; for a systematic review, refer to Almosa et al. (2017a) and Singh and Kaur (2021)], most studies focused on unilevel factors, including examining the impact of one factor (e.g., individual cognitive factor, self-monitoring, Ojedokun and Balogun, 2013), and exploring the effectiveness and mechanism of one intervention (e.g., Wever et al., 2010; Bateson et al., 2013). However, littering, as a social problem, is complex and influenced by multiple interactive factors and it would be solved effectively when understanding the interactive mechanism of relevant factors (Carvalho and Mazzon, 2019). Understanding littering behavior incorporating multi-level factors is needed. In addition, a recent systematic review of litter reduction programs by Almosa et al. (2017a) suggested that there is still a lack of social marketing use in litter reduction efforts to date in developing countries.

Vietnam is one of the most affected countries by environmental pollution and littering (Hoang et al., 2019). According to statistics from the Ministry of Natural Resources and Environment, nearly 1.8 million tonnes of trash is produced every year in Vietnam and this amount is predicted to increase in the next few years (Ministry of Natural Resources and Environment, 2019). Vietnam has taken actions to promote environmental protection and littering reduction (Thanh and Matsui, 2013; Schneider et al., 2017). For example, the Vietnam government has adopted legal documents regarding environmental protection and management policies, including the *Law of Environment Protection 2014* and *Decree NO. 38/NÐ-CP ON* the control of wastes. There is also a fine policy about littering that imposes heavy fines of up to VNÐ 7 million (USD 301) on those who litter in public places (Vietnam News, 2018). Furthermore, the Vietnam non-government organizations (NGOs) also took actions to raise public awareness and influence behavioral change and littering, such as the events *Clean-up and Recycling Day* and *Working with Fisherman* organized by Greenhub (Nguyen et al., 2015). However, the reported behavioral change is meager (Tuoitrenews, 2015). Previous studies have demonstrated that policy or a campaign delivered without understanding what the public wants and needs creates resistance (Pang and Kubacki, 2015). Therefore, it is necessary to solve the littering problem in Vietnam with social marketing—understanding the Vietnamese littering behavior and designing effective interventions and/or campaigns to promote voluntary change.

Conducting formative research with theories helps to understand a behavior (Stead et al., 2007; Brown et al., 2010). However, using one theory always could not fully explain a complex behavior, while using multiple theories or theoretical contracts is better to explain or predict one behavior (Carvalho and Mazzon, 2019). For example, the theory of planned behavior (TPB), which argues that behavioral intention is determined by attitude, perceived behavioral control (PBC), and social norms (Ajzen, 1991), is one of the widely used theories to explain human behavior (Leeuw et al., 2015). However, social norms are always examined as injunctive and descriptive norms (e.g., Pang et al., 2018), which come from the theory of normative social behavior (Rimal, 2008). In addition to intrinsic factors, the extrinsic nature- or environment-related variables (Deci and Ryan, 2000), particularly emotional connectedness to nature and environmental knowledge, were incorporated into the TPB and were found could better explain or predict pro-environmental behavior (Clark et al., 2019), such as protecting the remnant vegetation (Gosling and Williams, 2010) and tourists bringing litter down the mountain (Hu et al., 2018). It is worth noting that environmental knowledge is the cognitive dimension of environmental consciousness (Lafuente and Sanchez, 2010; Sharma and Bansal, 2013), which has not been examined for predicting littering behavioral intention. In addition, previous studies that added variables to TPB still examined them in an extended TPB framework, which did not distinguish the level of these factors (Yadav and Pathak, 2016; Hu et al., 2018; Ibrahim et al., 2021). This manuscript, thus, explores the factors influencing Vietnamese littering behavior by incorporating connectedness to nature and environmental consciousness into the TPB. When considering all the individual-, social-, and environmental-level factors, the multi-level social ecological model (SEM) is needed as a theoretical framework to guide model building and intervention development (Almosa et al., 2017b).

Based on the above practical limitations and research gaps, the purpose of this research is 3-fold. First, this study aims to insight into the littering problem in Vietnam by predicting Vietnamese intention to stop littering. Second, this study aims to explore the multi-level factors affecting Vietnamese littering intention by incorporating emotional connectedness to nature and environmental consciousness with the individual cognitive and social factors in the TPB. Third, this study adopted SEM to guide model building with all the individual-, social-, and environmental-level factors. Extensions and modifications will be made to the original model to provide any specificity on the context, which will also contribute to the literature in the areas of behavioral change and social marketing. The results show that individual cognitive PBC and emotional connectedness to nature are the two main factors predicting Vietnamese intention to stop littering, while environmental consciousness does not. Attitude is not always a predictor of Vietnamese intention to stop littering because of the significant moderating effect of injunctive and descriptive norms. The insights into Vietnamese littering behavior will provide implications to improve the effectiveness of existing policies and campaigns and/or to develop new policies and marketing campaigns to reduce littering behavior in Vietnam.

## Literature review and hypotheses development

### Littering behaviors and solutions

Littering is defined as individuals’ careless and incorrect disposal of minor amounts of waste at public venues such as on roads, at cafes, and in parks (Sibley and Liu, 2003). The waste is mainly paper, bottles, cigarette butts, food scraps, and plastic containers that are small but often have a significantly negative impact on the environment and society (Torgler et al., 2012). Since the 1970s, numerous scholars have called for the urgency of understanding the mechanism behind littering behavior (Krauss et al., 1978; Cialdini et al., 1990). To date, the literature has reached a few general agreements. For example, many researchers have found that littering is influenced by situational factors such as proximity/availability of trash bins (e.g., Schultz et al., 2013; Rasool et al., 2022) or the presence of companion (e.g., Hu et al., 2018), or by the individual demographic characteristics (e.g., age, gender, and education level) (Ojedokun and Balogun, 2013). However, littering would be not situational when the cognitive/psychological tendency and/or social norms are strong enough (Ojedokun, 2011; Ong and Sovacool, 2012). For example, the Japanese always do not litter in public places whatever the scenario (Ong and Sovacool, 2012). Besides, littering researchers also identified the impact of cognitive factors (e.g., individual cognitive factors, self-monitoring, Ojedokun and Balogun, 2013) and social factors (e.g., injunctive and descriptive norms, Bateson et al., 2015). However, most research focused on unilevel factors (e.g., Farage et al., 2021). Littering, as a social problem, is complex and influenced by multiple interactive factors and it would be solved effectively when understanding the interactive mechanism of relevant factors (Carvalho and Mazzon, 2019). Therefore, understanding littering behavior incorporating multi-level factors is needed.

### Individual cognitive factor: Perceived behavioral control

Perceived behavioral control refers to the perceived ease or difficulty of performing the behavior and is determined by control beliefs about the behavior (Ajzen, 1991). According to Kals and Maes (2002), control beliefs are one significant cognition that predicts sustainable behavior. Thus, the PBC is an important cognitive factor that could predict pro-environmental behavioral intention, including recycling, conservation, littering, and many other pro-environmental behaviors (Lokhorst et al., 2014; Leeuw et al., 2015; Hu et al., 2018). For example, Hu et al. (2018) found that tourists’ PBC significantly affects their litter management behavioral intention. Moreover, the attitudinal component is directly related to intention (Kelly and Breinlinger, 1995) and the attitude toward behavior mediates the relationship between PBC and behavioral intention (Hu et al., 2018). Since littering has a negative impact on the environment and society and intention is a strong predictor for the actual behavior (Ajzen, 1991), researchers have examined the attitude toward stopping littering and the intention to stop littering to predict behavioral change (Ojedokun and Balogun, 2013). Thus, we propose the following hypotheses:

**H1:** Perceived behavioral control impacts the intention to stop littering through a partial mediating effect of attitude toward stopping littering.

**H1.1:** Perceived behavioral control has a positive influence on the attitude toward stopping littering.

**H1.2:** Perceived behavioral control has a positive influence on the intention to stop littering.

**H1.3:** Attitude toward stopping littering has a positive influence on the intention to stop littering.

### Individual emotional factor: Connectedness to nature

Previous studies suggested that people-environment relations might play an important role in ecological behavior (Lokhorst et al., 2014). Connectedness to nature refers to one’s connection with the natural world (Andrews, 2018). Capaldi et al. (2014) regarded connectedness to nature as a personality construct that reflects individual differences in cognitive, affective, and experiential connection with the natural environment, and found a relationship between subjective nature connectedness and happiness. However, many other researchers approved its psychometric properties and treated it as an individual’s trait level of emotional connection to the natural world (Mayer and Frantz, 2004). From this perspective, connectedness to nature was found to be a strong predictor of pro-environmental attitudes and behaviors (Gosling and Williams, 2010; Lokhorst et al., 2014). For example, Gosling and Williams (2010) found that connectedness to nature has a positive effect on a farmer’s vegetation protection behavior. Lokhorst et al. (2014) found that the more landowners feel connected to nature, the higher their attitude and intention to conserve it. Reducing/stopping littering is good behavior for the natural environment (World Bank Group, 2017). Thus, we propose the following hypotheses:

**H2:** Connectedness to nature impacts the intention to stop littering through the partial mediating effect of attitude toward stopping littering.

**H2.1:** Connectedness to nature has a positive influence on attitude toward stopping littering.

**H2.2:** Connectedness to nature has a positive influence on the intention to stop littering.

### Social factors: Injunctive and descriptive norms

Littering, a behavior taking place in public places, is influenced by social pressure (Brown et al., 2010). One’s psychological decision-making process about littering is affected by whether the behavior is approved by the society in which they live, an injunctive norm, or how most others behave, a descriptive norm (Hu et al., 2018). To date, many researchers have examined the influence of injunctive and descriptive norms on people’s behavioral intentions (Leeuw et al., 2015). For example, Smith et al. (2012) found that injunctive and descriptive norms could interact to influence people’s environmental intentions. Furthermore, several studies suggested the moderating role of injunctive and descriptive norms (Liang and Shiau, 2018). For example, Liang and Shiau (2018) found the moderating effects of injunctive and descriptive norms on the relationship between customer satisfaction and repurchase intention. Fleury and Lee (2006) also found that where community members are provided with examples of success, social norms could enhance efficacy in goal formation and motivation related to physical activity. According to the social-ecological model, injunctive and descriptive norms are social/interpersonal factors, which are at a higher level than intrapersonal factors and influence individuals’ psychological decision-making process (Almosa et al., 2017b). The higher one’s injunctive and descriptive norms, the less the effects of individuals’ factors on their behavioral intention. In the littering context, injunctive and descriptive norms would moderate the formation of one’s littering intention. Thus, we propose the following hypotheses:

**H3:** Injunctive norms have a moderating effect on the whole model.

**H4:** Descriptive norms have a moderating effect on the whole model.

### Environmental consciousness

In the pro-environmental context, many studies have found that environment-related variables, such as environmental information/knowledge, environmental beliefs/values, and environmental awareness/concern, play an important role in predicting behavior or behavioral intention. Lafuente and Sanchez (2010) and Sharma and Bansal (2013) argued that these are cognitive and affective dimensions of environmental consciousness. Environmental consciousness is a general concept that refers to the degree to which a person is oriented toward concern for the environment (Lin and Chang, 2012). Many studies found a significant direct effect of environmental consciousness and/or its different dimensions (e.g., environmental knowledge) on pro-environmental behavior or behavioral intention (Hu et al., 2018). Besides, researchers also supported the moderating role of environmental consciousness in predicting pro-environmental behavior or behavioral intention (Lin and Chang, 2012; Leaniz et al., 2018). For example, Leaniz et al. (2018) found that environmental consciousness moderates the relationship between the green attributes of a hotel and customer perception of the hotel’s green image, which will promote customers. Environmental consciousness is related to one’s personal values (Lafuente and Sanchez, 2010). The more one perceives themselves as environmentally conscious, the more other attributes will influence their pro-environmental behavior or behavioral intention. In turn, that will satisfy their personal values (Lin and Chang, 2012). Thus, we propose the following hypothesis:

**H5:** Environmental consciousness has a moderating effect on the whole model.

**FIGURE 1:**
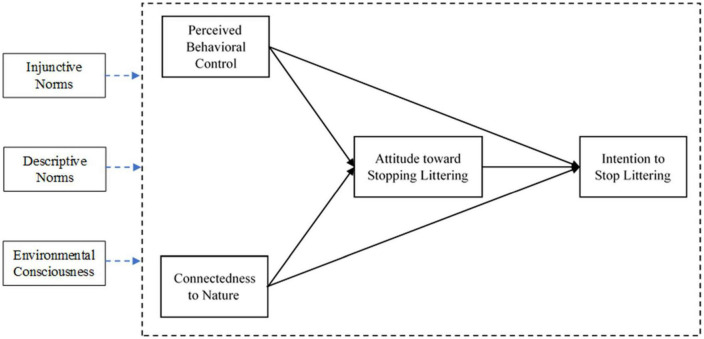
Conceptual model for explaining littering behavior.

In summary, we propose the following modified version of a social ecological model to explain the intention to stop littering (Figure 1).

## Materials and methods

### Data collection

The questionnaire was originally developed in English and translated to Vietnamese, and then back-translated to ensure consistency by two bilingual experts to ensure the meanings were consistent. Qualtrics was used to design the online questionnaire. Data were collected in Hanoi, the capital of Vietnam. Given the results of many field experiments which suggested that young people usually litter more than older (Ojedokun and Balogun, 2013) and the fact that students usually respond to and fill out the questionnaire positively and seriously (Bateson et al., 2013), this study selected students as potential participants. In addition, considering that people below 18 years might not fully form their own beliefs and/or values, we intended to only collect responses from adults. Taking together, the students in Hanoi above 18 years of age were selected as a sample to represent the Vietnamese. For convenience sampling, an anonymous link to the questionnaire was posted in Facebook groups of Vietnamese students in Hanoi. Two funneling questions, “Are you over 18 years old?” and “Do you live in Hanoi?” were placed at the beginning of the online survey to ensure the eligibility of the respondents. The ones who did pass the two questions would go to the end of the survey. In total, 623 valid responses were finally obtained to do data analysis.

### Measurements

All the variables were measured using 7-point Likert scales. Connectedness to nature items was adopted by Mayer and Frantz (2004). The PBC and attitude items were adopted from Fielding et al. (2008). The injunctive norms were adopted from Fielding et al. (2008) and Huber et al. (2018), and the descriptive norms were adopted from Fielding et al. (2016). The environmental consciousness scale was adopted by D’Amico et al. (2016). Intention to stop littering was used to measure behavioral intention because of the negativity of littering and the scale items were adopted from Rundle-Thiele et al. (2013).

### Data analysis

IBM SPSS 26.0 was used to analyze the descriptive statistics of the sample. Partial Least Squares Structural Equation Modeling (PLS-SEM) was then employed to examine the measurements and structural model using SmartPLS 3.0. Employing a component-based approach for estimation purposes (Lohmöller, 1989), the PLS-SEM is an alternative method to covariance-based SEM (usually AMOS) but it can be used with fewer indicators (1 or 2) per construct. PLS is better suited for explaining complex relationships because it avoids two problems: inadmissible solutions and factor indeterminacy (Fornell and Bookstein, 1982). Thus, this research adopted PLS-SEM to accommodate the two indicator variables (descriptive norms) and moderating effects.

There is three multi-group analysis (MGA) approaches Parametric Test, Welch–Satterthwait Test, and Henseler’s approach (Sarstedt et al., 2011). The parametric approach that initially was proposed by Keil et al. (2000) adopts the equal variances standardized *t*-test. The data provided should achieve the requirement of normal distribution, which does not fit the PLS path modeling’s distribution-free character. Chin et al. (2003) modified it by performing the unequal variance of standardized *t*-test (Welch–Satterthwait), which is more conservative but always shows the same testing results as parametric. Henseler’s PLS MGA is the most conservative approach (Henseler, 2007). Overall, the parametric test is the most widely used method (Sarstedt et al., 2011). Therefore, this research reports the *p*-value of the parametric, together with the Path Coefficients difference and *t*-value, to examine each of the proposed hypotheses.

## Results

### Respondents’ profiles

Table 1 shows the demographic characteristic of the respondents. All respondents were over 18 years old with a majority aged between 18 and 25 years. Almost all the respondents had attended university (97.7%). Respondents had a monthly income of around AU$50–AU$300 (74.9%). The majority were women (80.6%).

**TABLE 1:** Respondents’ demographic statistics.

Variable (N)	Category	n	Valid (%)
**Age (610)**			
	18–20 years old	413	67.7
	21–25 years old	194	31.8
	26–35 years old	3	0.5
**Education status (612)**			
	High school or below	12	2.0
	Diploma	1	0.2
	Some college	1	0.2
	University	596	97.4
	Master or higher	2	0.3
**Monthly income (411)**			
	Below $50	44	10.7
	$50–$300	308	74.9
	$301–$500	28	6.8
	More than $500	6	1.5
	Deny answer	25	6.1

The number in each variable is slightly different because of missing data.

### Measurement model

A reliability and validity analysis was conducted to assess the adequacy of the measurement model. The indicator reliability was determined by loadings and the results suggested a high degree of individual item reliability of 0.7 (Nunnally, 1978). Composite reliability (CR) and Cronbach’s alpha were both used to measure the internal consistency reliability of the items in terms of a unidimensional construct as suggested by Hulland (1999). All constructs achieved acceptable or excellent reliability (all between 0.71 and 0.98, refer to Table 2). The convergent validity was assessed through the average variance extracted (AVE). All AVE values were above the acceptable value of 0.5 (Fornell and Larcker, 1981; Bagozzi and Yi, 1988).

**TABLE 2:** Results of the measurement model.

Constructs/Items	Items (7-point scale)	Loadings	Cronbach’s α	AVE^a^	CR^b^
Perceived behavioral control			0.714	0.536	0.821
PBC1	If I wanted to, I could easily stop littering in public place.	0.781			
PBC2	It is mostly up to me whether I stop littering in public place.	0.778			
PBC4	How difficult would it be for you to stop littering in public place?	0.671			
PBC5	How much control do you have over whether you stop littering in public place?	0.692			
Connectedness to nature			0.841	0.676	0.893
CtN2	I often feel that I am a part of nature.	0.825			
CtN3	I often feel close to the natural world around me.	0.843			
CtN5	My own welfare is linked to the welfare of the natural world.	0.802			
CtN6	I recognize and appreciate the intelligence of other living things.	0.819			
Attitude toward stopping littering			0.984	0.926	0.987
Att_SL1	I think that littering in public place is: [Bad-good].	0.950			
Att_SL2	I think that littering in public place is: [Foolish-wise].	0.951			
Att_SL3	I think that littering in public place is: [Harmful-beneficial].	0.977			
Att_SL4	I think that littering in public place is: [Unpleasant-pleasant].	0.970			
Att_SL5	I think that littering in public place is: [Unsatisfying-satisfying].	0.954			
Att_SL6	I think that littering in public place is: [Unfavorable-favorable].	0.971			
Intention to stop littering			0.749	0.666	0.857
Int_SL1	I plan to stop littering in public place during the next 12 months.	0.796			
Int_SL2	I will stop littering in public place in the next 12 months.	0.795			
Int_SL3	I intend to stop littering in public place over the next 12 months.	0.856			
Injunctive norms			0.777		
INs3	If I litter in public place, people who are important to me would: [Completely disapprove-completely approve].	0.594			
INs4	Most people who are important to me think that littering in public place is: [Completely undesirable-completely desirable].	0.773			
INs5	Most people who are important to me think that [I should not-I should] littering in public place.	0.741			
Descriptive norms					
DNs1	Most members of my community currently stop littering in public place.	0.687			
DNs2	How much agreement is there amongst members of your community that stop littering in public place is a good thing?	0.689			
Environmental consciousness			0.812		
EC1	I plan to protect environment during the next 12 months.	0.640			
EC2	I will protect environment in the next 12 months.	0.756			
EC3	I intend to protect environment over the next 12 months.	0.787			

Items removed: indicator loadings are below 0.5: PBC3; CtN1, 4, 7; INs1, 2; DNs3.

^a^AVE, average variance extracted.

^b^CR, composite reliability.

### Structural model

Bootstrapping analysis was used to evaluate the direct and indirect effects of all hypothesized relationships using SmartPLS 3.0. Table 3 shows the results. The hypotheses testing results showed that PBC had a significantly positive effect on attitude toward stopping littering (*β* = 0.180, *t* = 4.395, *p* < 0.001) and intention to stop littering (*β* = 0.390, *t* = 8.385, *p* < 0.001). However, the results indicated the attitude toward stopping littering was not significantly related to the intention to stop littering (*β* = 0.026, *t* = 0.629, *p* > 0.1). Thus, H1.1 and H1.2 were supported, whereas H1.3 was not supported. Furthermore, the indirect effect of PBC on behavioral intention through attitude was not significant (*β* = 0.004, *t* = 0.593, *p* > 0.1) and therefore did not support H1. In addition, the results suggested that connectedness to nature was positively related to the intention to stop littering (*β* = 0.252, *t* = 5.991, *p* < 0.001) but was not significantly related to attitude toward stopping littering of general trash (*β* = 0.034; *t* = 0.849, *p* > 0.1). Thus, H2.1 was not supported, whereas H2.2 was supported. Furthermore, the indirect effect of connectedness to nature on behavioral intention through attitude was not significant (*β* = −0.001, *t* = 0.356, *p* > 0.1) and did not support H2.

**TABLE 3:** Results of the structural model.

	Path	*β*	S. E	|T-value|^∧^	95% CI LL	95% CI UL	Decision
H1.1	PBC → Att_SL	0.180	0.040	4.395***	0.115	0.248	Supported
H1.2	PBC → Int_SL	0.390	0.046	8.385***	0.313	0.463	Supported
H1.3	Att_SL → Int_SL	0.026	0.040	0.629	–0.092	0.041	Not supported
H1	PBC → Att_SL → Int_SL	0.004	0.007	0.593	–0.008	0.017	Not supported
H2.1	CtN → Att_SL	–0.034	0.041	0.849	–0.036	0.100	Not supported
H2.2	CtN → Int_SL	0.252	0.042	5.991***	0.186	0.325	Supported
H2	CtN → Att_SL → Int_SL	–0.001	0.002	0.356	–0.005	0.002	Not supported

PBC, perceived behavioral control; Att_SL, attitude to stop littering; Int_SL, intention to stop littering; CtN, connectedness to nature.

***p < 0.001.

### Moderation

This study considers injunctive norms, descriptive norms, and environmental consciousness as moderators of the relationships in the proposed model. For each moderator, we first computed the mean-value using SPSS and then separated the sample into two groups: high and low. For the descriptive norms, for example, the records whose value was higher than the mean (M_DNs_ = 3.22) were added to the high descriptive norms group (*n* = 293), and the records whose value was lower than the mean were added to low descriptive norms group (*n* = 330). Multi-group analysis (MGA) was then conducted to analyze the moderating effects using SmartPLS 3.0 as shown in Table 4.

**TABLE 4:** Results of multi-group analysis.

		High group			Low group			High vs. low group		
		*β*1	*t*	*P*	*β*2	*t*	*P*	Δ*β*	*t*	*P*
H3	PBC → Att_SL	0.14	1.91	0.06	0.24	3.67	0.000	0.10	1.01	0.31
INs	**PBC → Int_SL**	0.27	4.25	0.000	0.53	8.51	0.000	**0.26**	**2.79**	**0.005**
	Att_SL → Int_SL	−0.01	0.08	0.94	0.04	0.62	0.53	0.05	0.61	0.54
	CtN → Att_SL	−0.05	0.84	0.40	−0.04	0.50	0.62	0.01	0.14	0.89
	CtN → Int_SL	0.27	4.36	0.000	0.22	3.49	0.001	0.04	0.46	0.65
	n	361			262					
H4	PBC → Att_SL	0.21	3.40	0.001	0.17	2.56	0.011	0.05	0.61	0.54
DNs	PBC → Int_SL	0.48	7.43	0.000	0.33	5.32	0.000	0.16	1.77	0.08
	**Att_SL → Int_SL**	−0.07	1.47	0.14	0.13	2.14	0.03	**0.20**	**2.67**	**0.008**
	CtN → Att_SL	0.00	0.08	0.94	−0.05	0.79	0.43	0.04	0.49	0.62
	**CtN → Int_SL**	0.16	2.79	0.005	0.34	6.23	0.000	**0.18**	**2.29**	**0.02**
	n	293			330					
H5	PBC → Att_SL	0.19	2.84	0.005	0.18	2.80	0.005	0.00	0.01	0.99
EC	PBC → Int_SL	0.38	5.34	0.000	0.41	6.27	0.000	0.03	0.29	0.78
	Att_SL → Int_SL	0.03	0.68	0.50	0.02	0.37	0.72	0.01	0.14	0.89
	CtN → Att_SL	0.11	1.17	0.24	−0.13	1.98	0.04	0.25	1.84	0.07
	CtN → Int_SL	0.05	0.59	0.56	0.28	4.79	0.000	0.23	1.82	0.07
	n	351			272					

Table in bold indicate the significance of multi-group analysis.

The results showed that the path coefficients between PBC and intention to stop littering had a significant difference between the high injunctive norms group and the low injunctive norms group (Δ*β* = 0.26, *t* = 2.79, *p* < 0.01). More specifically, the strength of the relationship for the high injunctive norms (*β*_L–INs_ = 0.53, *t* = 8.51, *p* < 0.001) between PBC and intention to stop littering was significantly greater than for the low injunctive norms (*β*_H–INs_ = 0.27, *t* = 4.25, *p* < 0.001). This result indicated that for Vietnamese who feel most others’ disapproval of littering, individuals’ PBC had a greater effect on behavioral intention to stop littering than Vietnamese who feels the society’s approval of littering. Therefore, H3 was supported.

The results also suggested the moderating role of descriptive norms. On one hand, the strength of the relationship between connectedness to nature and intention to stop littering had a significant difference between the high and low descriptive norms group (Δ*β* = 0.18, *t* = 2.30, *p* < 0.05). More specifically, the strength of the relationship for the low descriptive norms (*β*_L–DNs_ = 0.34, *t* = 6.23, *p* < 0.001) between connectedness to nature and intention to stop littering was significantly greater than for the high descriptive norms (*β*_H–DNs_ = 0.16, *t* = 2.79, *p* < 0.01). On the other hand, the path Coefficients between attitude and intention to stop littering had a significant difference between the high and low descriptive norms group (Δ*β* = 0.20, *t* = 2.67, *p* < 0.01). More specifically, for the Vietnamese who found most others in the community littering, the attitude toward stopping littering had a significantly positive effect on the intention to stop littering (*β*_L–DNs_ = 0.13, *t* = 2.14, *p* < 0.05), while the attitude could not predict behavioral intention to stop littering for those Vietnamese who found most others do not litter (*β*_H–DNs_ = −0.07, *t* = 1.47, *p* > 0.05). Thus, H4 was supported.

**FIGURE 2:**
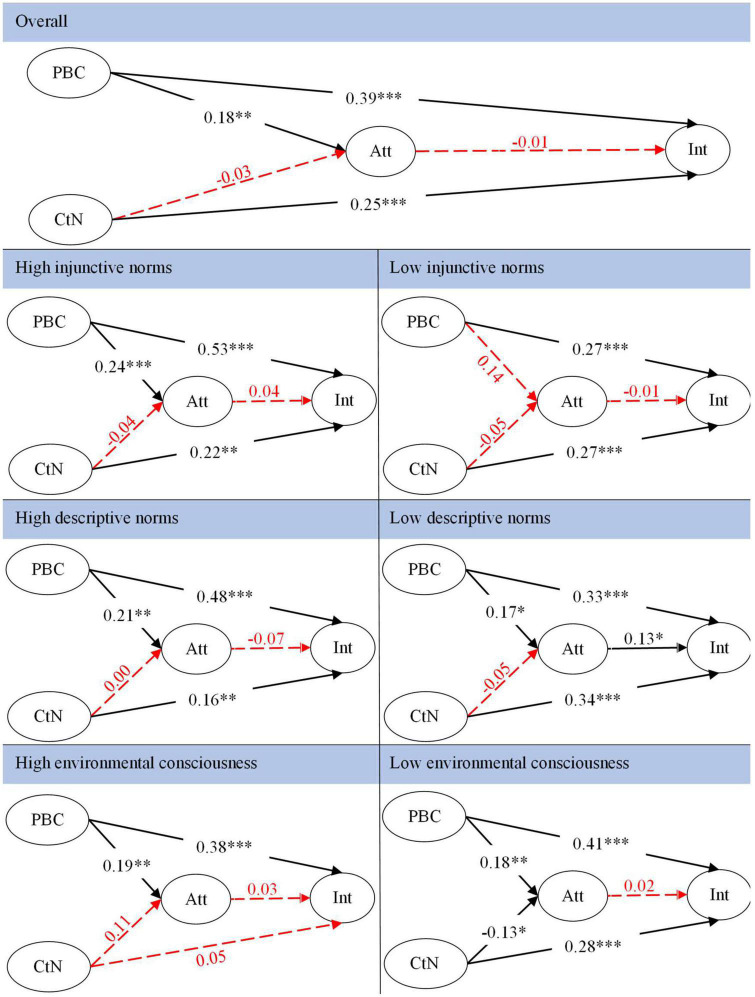
The measurement model overall and for high/low injunctive norms, descriptive norms, and environmental consciousness. *p < 0.05, ^**^p < 0.01, and ^***^p < 0.001.

In addition, the results also showed the different effects of connectedness to nature on the intention to stop littering between different environmental consciousness groups. More specifically, for the Vietnamese who were more concerned about the environment, the connectedness to nature had a significantly positive effect on the intention to stop littering (*β*_L–EC_ = 0.28, *t* = 4.79, *p* < 0.001), while this effect was not significant for the Vietnamese who were less concerned about the environment (*β*_H–EC_ = 0.05, *t* = 0.59, *p* > 0.05). Overall, the relationship between connectedness to nature and behavioral intention was not significant (Δ*β* = 0.23, *t* = 1.82, *p* > 0.05). Thus, H5 was not supported. For more details please refer to Figure 2.

## Discussion and conclusion

This study sought to understand the littering behavior of Vietnamese by incorporating emotional connectedness to nature and environmental consciousness into the TPB, which focuses on cognitive and social factors. We also adopted the SEM to guide model building due to the multi-level factors. The results showed that PBC and connectedness to nature have significantly positive influences on Vietnamese intention to stop littering, whereas attitude toward stopping littering could not predict Vietnamese intention to stop littering effectively. Surprisingly, we found that injunctive and descriptive norms have moderating effects. The following section of this article discusses the results in relation to previous studies and social marketing’s potential to prevent littering.

### Factors influencing Vietnamese intention to stop littering

Perceived behavioral control and connectedness to nature had significant positive influences on Vietnamese intention to stop littering, while attitude toward stopping littering could not predict Vietnamese intention to stop littering. This was partly inconsistent with previous research, Lokhorst et al. (2014) found that attitude, PBC, and connectedness to nature are all the key factors impacting pro-environmental behavioral intention, more specifically the nature conservation intention. The positive impact of connectedness to nature on intention to stop littering is similar to and extends previous research that identified the influence of connectedness to nature on behavioral intention in the context of reducing plastic use (Clark et al., 2019) and vegetarian protection (Gosling and Williams, 2010). Our finding that attitude toward stopping littering had no effect on the intention to stop littering is different from Ojedokun and Balogun’s (2013) result that attitude toward littering significantly impacts responsible environmental behavior. In addition to social norms, another possible explanation for this finding is the situational limitation, the high prevalence of littering behavior, and the low availability of trash bins in Vietnam, which limits the conversion of attitude toward stopping littering to behavior. Thus, in order to prevent the earth/globe (e.g., the environment, health, biodiversity, and even economy) from the negative effects of littering, more social marketing research in different contexts are needed to explore the factors influencing littering behavior and/or behavioral intention and further to guide targeted interventions to promote behavior change.

### The moderating role of injunctive norms

Injunctive norms were found to moderate the relationship between PBC and the intention to stop littering. This is different from the results of most previous studies (e.g., Hu et al., 2018; Clark et al., 2019) where injunctive norms and PBC are treated equally as the independent predictors of pro-environmental behavioral intention. Based on the MGA results, the influence of PBC on intention to stop littering for high injunctive norms was greater than for low injunctive norms. That is, the injunctive norms had a positive moderating effect on the behavioral intention about littering. This is different from Stok et al.’s (2014) results that injunctive norms had a negative effect on behavioral intention. Probably because the behavior in Stok et al.’s (2014) study is promotional behavior (fruit taken), for which high injunctive norms would lead people to feel like they are limited freedom of acting and, thus, induce resistance to the proposed behavior (Stok et al., 2014). However, the behavior in our study is preventive (littering), for which higher injunctive norms would make people feel stronger disapproval of the unwanted behavior and thus act in the proposed direction. More social marketing research should be conducted to further explore the injunctive norms’ role and effect on various promotional and preventive behaviors. With the formative research results, designing corresponding interventions to promote positive behavior and prevent negative behavior change.

### The moderating role of descriptive norms

The descriptive norms had a significant moderating effect on the relationship between connectedness to nature and intention to stop littering, and the relationship between attitude and intention to stop littering. This strengthens previous results that descriptive norms play an important role in explaining littering behavior (Bateson et al., 2013) and other pro-environmental behaviors (e.g., Leeuw et al., 2015). Besides, by distinguishing individual- and social-level factors and building a theoretical research model with SEM, we found that the descriptive norms negatively moderate the effect of connectedness to nature on the intention to stop littering. This confirms some recent opinions (e.g., Carvalho and Mazzon, 2019) that social problems are complex and often involve multiple interacting factors. Therefore, in order to solve the littering problem, interventions and/or campaigns should be designed beyond the traditional individual-focused approach and incorporate other social solutions such as promoting environmentalist models, encouraging citizen monitoring, as well as social norms approaches that can create a supportive interpersonal setting to facilitate behavioral changes.

In addition, the descriptive norms significantly moderate the relationship between attitude and intention to stop littering. This is different from most of the previous studies, where the attitude toward the behavior is always the strong predictor of behavioral intention (e.g., Kelly and Breinlinger, 1995; Leeuw et al., 2015). In our study, the attitude had a significantly positive impact on the intention to stop littering only for low descriptive norms but not for high descriptive norms. In particular, when one found most members of their community littering (low descriptive norms), the intention to stop littering was influenced by attitude, more specifically the PBC-attitude-intention indirect path. However, for the Vietnamese whose community members do not litter, their intention to stop littering was only directly influenced by PBC and connectedness to nature. Taking together, in a situation where most others littering, one may process more thoughts to determine their behavior and behavioral intention about littering. This finding reveals that Vietnamese are sensitive to and easily influenced by social cues. Future research could examine this with technology-based experiments, such as brainwave headsets.

### The influence of environmental consciousness

The results showed the difference between the high and low environmental consciousness group and their influence of connectedness to nature on the intention to stop littering, though the difference did not reach a significant level. Hu et al. (2018) regard environmental consciousness as a multi-dimensional variable and found that its cognitive dimension and environmental knowledge significantly impacted tourists’ litter management behavior down the mountain. The current study adopted unidimensional environmental consciousness and found that it had no significant effect on littering behavioral intention. One possible explanation is that tourists are always more sensitive to environmental variables (vs. individuals in the city) because of their higher affinity with the natural environment. Given those formative research, social marketing interventions and/or campaigns targeting littering reduction should be designed to influence individuals’ environmental knowledge. Differently, Leaniz et al. (2018) in the context of purchasing a green product, found the moderating effect of environmental consciousness. Thus, interventions targeting environmental consciousness would be effective for promoting responsible consumption but not for reducing or preventing littering. This finding also confirms the literature on the mixed effectiveness of the approach of education, targeting providing knowledge in the hope of changing behaviors. Education *per se* is not serving as the behavioral determinant but only provides knowledge which is only a moderating factor.

### Theoretical and practical implications

From the theoretical point of view, this study is the first to provide insight into littering behavior in Vietnam using a theoretical lens. Theory use has been scarce in the context of littering and previous research does not distinguish the level of factors influencing littering (e.g., Carvalho and Mazzon, 2019). In this study, we built a theoretical model by incorporating emotional connectedness to nature and environmental consciousness into the TPB and adopting the multi-level SEM’s principles. Therefore, this study contributes to the literature by providing a comprehensive dual-theory model with all the individual cognitive, social, and emotional nature- and environment-related factors. We found that both individuals’ cognition of PBC and emotional connectedness to nature play important roles in their littering intention. Moreover, we also found the moderating effect of injunctive norms on the relationship between PBC and behavioral intention, and the moderating effect of descriptive norms on the relationship between connectedness to nature and behavioral intention, and between attitude and behavioral intention. The findings extend our understanding of the nuisances of littering behavior in Vietnam by disentangling the interactive mechanism of multi-level factors. Overall, this study offers a new research perspective for understanding littering behavior. Such insights can be directly translated into actionable solutions to inform and help future social marketing campaign designs in order to tackle the littering issue.

From a practical review, the results of this study provided some directions for government or social marketing practitioners and even managers to take action to eliminate/reduce littering in Vietnam through three social marketing activities (which are regarded as “products” in the context of 4Ps in social marketing). *First*, the current study clearly indicated that individuals’ cognition of PBC had significantly positive effects on Vietnamese intention to stop littering. Therefore, improving Vietnamese PBC would be an effective way to reduce littering behavior and then environmental pollution. It can be achieved by displaying verbal prompts, such as “It is easy to stop littering” or “You can control not littering” over the walls, lawns, trash bins, product packages, or digital platforms, which might improve the effectiveness of existing strategy such as providing more trash bins. Displaying these prompts should be performed consistently across urban and rural areas in Vietnam to increase effectiveness, and these campaigns should be promoted through mass media and social media to enhance Vietnamese awareness. Moreover, the government could mobilize citizens to co-design and vote for the displayed prompts and to disseminate the campaign with digital channels (Abbate et al., 2022), through which people’s PBC and further the intervention’s effectiveness would be enhanced (Jong et al., 2019).

*Second*, the current study clearly indicated that individuals’ intention to stop littering increases with their emotional connectedness to nature. Clark et al. (2019) found that nature-based experiences contribute to connectedness to nature. In Vietnam, the existing initiatives organized by the NGOs, such as *Clean-up and Recycling* and *Working with Fisherman* could provide this kind of experience, but the participants are usually volunteers who might already have high connectedness to nature and not litter in public places (Nguyen et al., 2015). Therefore, to successfully promote a littering reduction in the whole of Vietnam, activities should be re-designed to attract and suit everyone’s participation. For example, some programs which can help to build up the connectedness to nature such as sea kayaking, tour aquarium, mountain climbing, spring, and autumn nature tour, and other outdoor activities should be promoted by every school, company, and community to every child, student, worker, and citizen. Through such programs, more connections with nature can be built. During the programs, the detrimental effects caused by littering can be highlighted through digital storytelling to increase awareness to protect the environment (Andriopoulou et al., 2022). The cost for these programs should be low or reasonable to ensure that low-income people would not be disadvantaged by their limited income. These programs should be promoted through all the media channels of schools, companies, and communities to reach wide participants.

*Third*, the results suggest that a focus on descriptive norms may improve the effectiveness of relevant campaigns or interventions targeting individual cognition of PBC and emotional connectedness to nature. As indicated by the results of this study, PBC has a more significant influence on the intention to stop littering when the Vietnamese hold high injunctive norms about littering. Therefore, in addition to trying to improve Vietnamese PBC, improving their perception of society’s disapproval of littering behavior would also be important. For this, social marketing practitioners could display a picture of watching eyes on the wall in public places to make people aware of injunctive norms regarding littering (Bateson et al., 2013). Littering penalties should be applied as well to show injunctive norms. Although Vietnam has applied some fine penalty policies, a heavier fine could prevent littering behavior. Community service litter clean up hours can be employed as a means of littering penalties. The government could pass a legal document to require the manufacturers to display the picture/text of anti-littering and putting litter into trash bins on product packages and in a conspicuous and prominent way. For this, manufacturing/corporate managers could invite their consumers to communicate and co-design the picture/text on a digital platform (e.g., brand community). This co-creation process would also help the manufacturers/corporates improve their reputation and enhance customer loyalty.

## Limitations and future research

The main limitation of the current study was the use of a self-report method to measure littering in public places. Reliance on this method for the wrongdoing of littering results in potential under-reporting due to socially desirable bias. In future research, objective measurements of actual littering behavior, such as observations or using global positioning system (GPS) technology and digital technologies (e.g., big data, artificial intelligence) could be adopted. Second, our study did not include any infrastructural factors, such as the level of pollution of places and the availability of trash bins. Broader theoretical perspectives are recommended for future research to comprehensively explain littering behavior. Third, this study only collected data from one city in Vietnam, which may limit the generalization of the results. Future research should use a Vietnam-wide sample to ensure the representativeness of the population to overcome this limitation. Finally, the survey was based only on students and most respondents were female, both of which were found related to the littering rate in some developed countries (e.g., the U.S.) (Krauss et al., 1978). Future research should use a more representative, larger scale sample with diverse demographic characteristics (education level, age, gender) to re-examine the psychological and social factors influencing Vietnamese littering intention so as to draw a more rigorous conclusion, and on the other hand, to explore the effects of demographic characteristics on Vietnamese littering rate and/or intention. Furthermore, future studies could adopt a longitudinal perspective of analysis. The interventions and campaigns, especially that on a digital platform or with digital channels, take time to be implemented and communication and knowledge learning happen during this time. Thus, longitudinal studies are needed to examine people’s knowledge, attitude, and practice (KAP) toward littering and other sustainable green behaviors/practices (e.g., waste separation and recycling) over time. Future research should also be able to introduce the temporal effects on expectant outcomes, given the costs associated with recycling behaviors are immediate whereas the returns are in the long term.

## Data availability statement

The raw data supporting the conclusions of this article will be made available by the authors, without undue reservation.

## Author contributions

All authors listed have made a substantial, direct, and intellectual contribution to the work, and approved it for publication.
